# Epidemiological characteristics of post-traumatic stress symptoms and its influence on length of hospital stay in inpatients with traumatic fractures in Zunyi, China

**DOI:** 10.3934/publichealth.2024042

**Published:** 2024-07-01

**Authors:** Guojia Qi, Xiu Dai, Xue Wang, Ping Yuan, Xiahong Li, Miao Qi, Xiuli Hu, Xiuquan Shi

**Affiliations:** 1 Department of Epidemiology and Health Statistics, School of Public Health, Zunyi Medical University, Zunyi, Guizhou, China; 2 Department of Endemic and Chronic Non-communicable Diseases Control, Huichuan District Center for Disease Control and Prevention, Zunyi 563000, Guizhou, China; 3 Key Laboratory of Maternal & Child Health and Exposure Science of Guizhou Higher Education Institutes, Zunyi, Guizhou, China; 4 Center for Injury Research and Policy & Center for Pediatric Trauma Research, The Research Institute at Nationwide Children's Hospital, The Ohio State University College of Medicine, Columbus, OH, USA

**Keywords:** traumatic fracture, epidemiology, length of hospital stay, post-traumatic stress symptoms, influencing factors

## Abstract

**Objectives:**

To investigate the clinical epidemiological characteristics and occurrence of post-traumatic stress symptoms (PTSS) in patients with traumatic fractures, we sought to analyze the factors that influence the prognosis of a length of hospital stay (LOS) and provide valuable insights to prevent PTSS in fracture patients and improve their prognosis.

**Methods:**

Inpatients with traumatic fractures were recruited from a third-class comprehensive general hospital in southwest China between November 2019 and October 2020. Case data of traumatic fracture patients were collected, and a questionnaire that included general information and basic fracture details was completed. The post-traumatic stress disorder Self-rating Scale was used to assess PTSS among the fracture inpatients.

**Results:**

A total of 204 inpatients who experienced traumatic fractures were included in this study. Falls accounted for the largest proportion of traumatic fractures. A Cox's regression analysis revealed that serious injury [Hazard Ratio (HR) = 2.44, 95% Confidence Interval (*CI*): 1.33–4.46], critical illness during hospitalization (HR = 1.70, 95% *CI*: 1.13–2.54), and undergoing two surgeries (HR = 1.87, 95% *CI*: 1.20–2.93) were risk factors for longer LOS. Among the fracture patients, 30.39% exhibited positive PTSD symptoms, and physical activity during the fracture [*Odds Ratio* (*OR*) = 0.63, 95% *CI*: 0.45–0.88] and increased pain (*OR* = 3.34, 95% *CI*: 1.82–6.11) were identified as influencing factors.

**Conclusions:**

Given the high detection rate of PTSS following traumatic fractures, it is crucial for relevant departments to implement targeted measures to protect high risk individuals. Furthermore, strengthening the care provided to the patients' physical and mental health is urgently needed to reduce the incidence of PTSS.

## 1. Introduction

Traumatic injuries are the leading cause of deaths and disabilities worldwide [1]. At the same time, they also pose a heavy burden for China [2]. In China, traumatic injuries have become the fifth most common cause of death, with the number of deaths exceeding those caused by diabetes and infectious diseases [3]. Moreover, fractures related to these injuries are the primary consumers of healthcare resources, posing tremendous pressure on the medical system. The average length of hospital stay (LOS) is about half a month, and 75.4% of the patients have a total hospitalization cost of more than $1539.55 [4],[5].

Traumatic fractures primarily arise from the impact of intense external forces on the body, resulting in the disruption of bone tissue. Such injuries are commonly seen in accidental situations such as traffic accidents, falls from heights, and slips. Among these, fractures in the limbs are particularly common [6],[7].

As a highly impactful stressor, traumatic fractures have profound and complex effects. They may either directly or indirectly induce a range of varying post-traumatic symptoms, such as insomnia, hypervigilance, fear, and avoidance [8],[9]. When these symptoms significantly impair mental functioning and persist for more than a month after the traumatic event, we tend to consider them as potential manifestations of post-traumatic stress disorder (PTSD) [10].

However, when the diagnostic criteria for PTSD are not met, it may be more appropriate to describe the patient's condition using the term “post-traumatic stress symptoms (PTSS)” [11]. Particularly during the acute phase of traumatic fractures, patients are prone to experiencing anxiety, depression, and significantly exacerbated fear and avoidance towards the trauma [8],[12]. Without timely attention and intervention, these symptoms are likely to gradually develop into PTSD [13].

A meta-analysis study has revealed that the combined incidence of PTSD among fracture patients is as high as 29% [9]. This figure further underscores the profound impact of traumatic fractures on a patients' mental health. Therefore, in the treatment and rehabilitation process of traumatic fractures, we must not only focus on the patient's physical recovery, but also prioritize the care of their mental health, ensuring that patients receive comprehensive attention and support in both physical and mental aspects.

Currently, our understanding of the associated risk factors between PTSS and fracture risk remains limited. Therefore, we have further investigated the epidemiological characteristics of patients with traumatic fractures and their relationship with PTSS, while delving into the relevant risk factors. The aim of this study is to provide a more solid theoretical basis for the early prevention of physical and mental health issues among patients with post-traumatic fractures to better safeguard their recovery and quality of life and minimize their hospital stay duration.

## 2. Methods

### 2.1. Participants

During the 6th National Population Census of Guizhou Province, the permanent resident population of Zunyi City was established as 6,127,082 individuals [14]. However, during the unique period from November 2019 to October 2020, due to the impact of the COVID-19 pandemic, we adopted a convenience sampling method to recruit patients with traumatic fractures from a third-class A comprehensive hospital in Zunyi City. This hospital, being the first “Advanced Trauma Center” in Guizhou Province, ensures that its patient population possesses a certain degree of representativeness.

To ascertain a suitable sample size for our research, we utilized Slovin's formula, which is defined as *n* = *N* / (1 + *N* × *e²*), where (*n*) indicates the sample number, (*N*) represents the total population, and (*e*) indicates the margin of error. With a 95% confidence interval (*CI*) and a 7% margin of error, the calculated sample size was estimated to be 204 individuals.

### 2.2. The inclusion and exclusion criteria

**Inclusion criteria:** 1) During the study period, patients with traumatic fractures were hospitalized in the emergency department and orthopedics department; 2) the patient possessed a clear consciousness and normal cognitive abilities, and was able to complete the questionnaire and related scales either independently or with the assistance of the investigator; and 3) the patient and/or their family members signed the informed consent form.

**Exclusion criteria:** 1) Patients with mental retardation, slurred speech, and communication disorders; 2) patients with a history of mental illness and disorders, or serious medical diseases before the injury; 3) patients with central nervous system disorders caused by acute trauma, with suffering from long-term or intermittent coma injuries and mental abnormalities; 4) those with fractures and traumatic brain injuries; and 5) patients with spontaneous fractures.

### 2.3. Data collection

During the patients' hospitalization, we collected case data from trauma patients with fractures who met the inclusion and exclusion criteria, and completed the relevant survey questionnaires. The questionnaires covered the patients' general information, basic information about their fractures, and possible acute stress symptoms of PTSD.

The post-traumatic stress disorder self-rating scale (PTSD-SS) is a self-reporting measure developed by Chinese experts Liu, et al. to capture the level of symptomatic responses to a specific traumatic stressor. The scale contains 24 items and 5 subscales, namely subjective assessment of traumatic events, intrusion, avoidance, hyperarousal, and impaired social function. The degree of distress for each item is rated on a 5-point scale, ranging from the absence of a symptom (score of 1) to maximal symptoms (score of 5). For symptoms of intrusion, avoidance, and hyperarousal, scores of each subscale were calculated and an average score above 2 was used as the cutoff value. A cut-score of 50 on the PTSD-SS total score indicated a clinically significant stress response. The higher the score, the more severe the stress reaction [15].

### 2.4. Definition of study variables

The etiology of the fracture was categorized as follows: traffic accident injuries, crush injuries, fall injuries from heights, machine injuries, life injuries (injuries caused by falls due to inadvertent movement), and other causes of fractures (attacked by others, sports sprain, natural disasters, etc.).

Surgery was defined as the various surgical operations experienced by the patient during hospitalization, including reduction, fixation, traction, and amputation. LOS was the real time interval between the date of admission and the date of discharge of the patient according to the medical record. The Abbreviated Injury Scale (AIS) was used to assess the severity of the injury [16]. Based on the anatomical classification system, the body can be divided into six parts: the head and neck, the chest, the face, the abdomen and pelvis, the limbs and pelvis, and the surface of the body.

The Injury Severity Score (ISS) [17] was derived from the sum of the squares of the highest AIS scores for three different body regions: minor injuries, ISS ≤ 16 points; severe injuries, ISS > 16 points; and severe injuries, ISS > 25 points. The mortality rate was significantly increased when ISS > 20 points, and there were few survivors when ISS > 50 points. When the AIS score of one or more body parts of the patient was 6, the ISS takes the highest score of 75.

The Numerical Rating Scale (Pain Measurement) (NRS) is a scale with 11 items, ranging from “0” (no pain) to “10 (severe pain): 0 means no pain, 1–3 point is mild pain, 4–7 point is moderate pain, and 8–10 point is severe pain.

### 2.5. Statistical analysis

Epidata 3.02 (EpiData Association, http://www.epidata.dk/, Denmark) was used to establish the database. The statistical analysis involved using SPSS 18.0 (IBM Corp., Armonk, NY, USA). The Chi-square test was used to compare the difference of different component ratios. An analysis of variance (ANOVA) and a t-test were used to compare differences in scores for PTSS across various clinical characteristics. The influencing factors of LOS and PTSS after fracture were analyzed by a multiple logistic regression and Cox's regression model, respectively. All tests were two tailed, and a *P* < 0.05 was considered as statistically significant.

## 3. Results

### 3.1. Demographic characteristics of hospitalized patients with traumatic fractures

**Figure 1.**
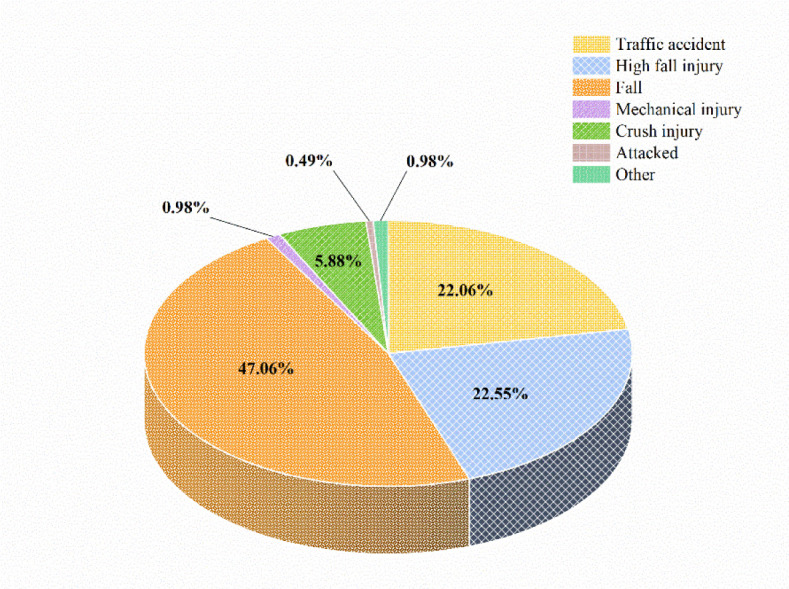
Distribution of etiology of traumatic fractures.

This study recruited 204 inpatients with traumatic fractures, with a male to female gender ratio of 1.52:1. Most patients were aged between 41 and 60, and over half of them came from rural areas. Occupationally, agricultural workers were the majority, and nearly half of the patients had only a primary school education or below. In terms of medical insurance, the urban residents' medical insurance accounted for the highest proportion (34.31%), followed by self-payment (28.92%). Upon discharge, 78.25% of the patients had limited self-care abilities (Table 1). A cause analysis revealed that falls (47.06%), falls from heights (22.55%), and traffic accidents (22.06%) were the main causes of traumatic fractures (Figure 1).

**Table 1.** General demographic characteristics of patients with traumatic fracture.

Variable	*N*	Percentage (%)
Gender		
Male	123	60.29
Female	81	39.71
Age, years		
≤20	7	3.43
21–40	45	22.06
41–60	83	40.69
61–80	59	28.92
>80	10	4.90
Residence		
City	50	24.51
Town	33	16.18
Countryside	121	59.31
Marital status		
Unmarried	17	8.33
Married	187	91.67
Ethnicity		
Han	174	85.29
Minority	30	14.71
Profession		
Agriculture	81	39.71
Business	16	7.84
Civil servants/Institutions	10	4.90
Workers/Enterprises	44	21.57
Retirement	9	4.41
Students	18	8.32
Unemployed	21	10.29
Other	5	2.45
Education degree		
Primary school or below	94	46.08
Junior school	50	24.51
High school	29	14.22
Junior college	18	8.82
Bachelor	13	6.37
Monthly income, yuan		
No	59	28.92
<2000	37	18.14
2000~	49	24.02
4000~	36	17.65
6000~	19	9.31
8000~	4	1.96
History of trauma		
Yes	56	27.45
No	148	72.55
Healthcare		
Staff	25	12.25
NCMS^a^	50	24.51
Urban residents^b^	70	34.31
Self-paying	59	28.92
Self-care after discharged		
Can not	10	4.90
Partly	150	73.53
Fully	44	21.57
Total	204	100.00

Notes: ^a^, new rural cooperative medical system; ^b^, urban residents' medical insurance system

### 3.2. Comparison of the etiology of fracture under different demographic and clinical characteristics

Our research indicates that male patients with fractures outnumbered females (*P* < 0.001). There exists an association between age and the cause of fractures (*χ²* = 8.27, *P* = 0.004, Pearson's *R* = 0.202).

Traffic accidents and falls from heights are mostly associated with multiple fractures, while falls are predominantly single fractures (*P* < 0.001). Varying causes of fractures can lead to significant differences in the severity of injury, pain, and comas (*P* < 0.05). (Table 2).

### 3.3. PTSS under different clinical characteristics of fractures

Among fracture patients in this study, 30.39% had positive PTSS. All differences were statistically significant for the following: the symptoms of re- experiences, the etiology of the fracture (*F* = 8.81, *P* < 0.001), the most severe site (*F* = 4.92, *P* < 0.001), the type of fracture (*F* = 19.32, *P* < 0.001), the severity of injury (*F* = 7.60, *P* = 0.001), activity during the fracture (*F* = 6.04, *P* < 0.001), comas (*F* = 11.90, *P* = 0.001), and the degree of pain (*F* = 13.25, *P* < 0.001).

In avoidance symptoms, the difference between the most severe site (*F* = 2.61, *P* = 0.019) and activity during the fracture (*F* = 3.35, *P* = 0.011) was statistically significant.

Moreover, all differences were statistically significant for the following: hyperarousal, etiology of fractures (*F* = 3.35, *P* = 0.02), the most severe site (*F* = 3.95, *P* = 0.001), the type of fracture (*F* = 13.71, *P* < 0.001), the severity of the injury (*F* = 6.18, *P* = 0.002), comas (*F* = 6.59, *P* = 0.011), and the degree of pain (*F* = 7.26, *P* < 0.001).

**Table 2.** Comparison of etiology of fractures in different gender, age and clinical characteristics.

Variable	Etiology of fractures				*χ^2^* value	*P*-value
	Traffic accident	High fall injuries	Fall	Other		
Gender					10.90	0.012
Male	27	35	48	13		
Female	18	11	48	4		
Age, years					8.27	0.004*
≤20	4	0	3	0		
21–40	12	10	18	5		
41–60	20	28	27	8		
61–80	9	8	38	4		
>80	0	0	10	0		
Location of fracture					165.92	<0.001
Home	0	10	54	1		
Community/School	1	4	18	3		
Streets/shopping malls	40	4	12	3		
Construction site	0	17	3	7		
wild	4	11	9	3		
Type of fracture					28.77	<0.001
Single fracture	16	13	66	5		
Multiple fractures	29	33	30	12		
Severity of injury^a^						<0.001**
Minor injuries	31	25	87	11	2.95	0.086*
Serious injuries	10	15	4	3		
Devastating injuries	4	6	5	3		
The degree of pain^b^					21.16	0.002
Mild	2	2	19	2	6.48	0.011*
Moderate	15	23	45	4		
Severe	28	18	30	11		
Coma						0.029**
Yes	7	4	6	5		
No	38	42	90	12		

Notes: *, Chi-square trend test; **, Fisher's Exact Test; ^a^, minor injuries, ISS≤16; serious injuries, ISS>16 and ≤25; devastating injuries, ISS>25; ^b^, mild pain, the score of Numerical Rating Scale (NRS) is 1–3, moderate pain is 4–7 and severe pain is 8–10.

The total score of the PTSS varies between groups, the etiology of the fracture (*F* = 4.59, *P* = 0.004), the most severe site (*F* = 4.56, *P* < 0.001), the type of fracture (*F* = 13.74, *P* < 0.001), the severity of the injury (*F* = 5.78, *P* = 0.004), activity during the fracture (*F* = 4.38, *P* = 0.002), comas (*F* = 5.29, *P* = 0.022), and the degree of pain (*F* = 8.77, *P* < 0.001) (Table 3).

**Table 3.** Scores of PTSS in the clinical fractures of characteristics (*Mean* ± *SD*).

Variable	Subjective assessment	Re- experiences	Avoidance	Hyperarousal	Damaged social function	Total score of PTSS
Etiology						
Traffic accident	2.80 ± 1.18	16.47 ± 5.53	11.89 ± 4.55	13.82 ± 3.87	4.16 ± 2.01	50.47 ± 15.14
High fall injury	2.54 ± 1.15	17.09 ± 5.38	11.50 ± 3.94	13.13 ± 3.80	3.54 ± 1.71	49.28 ± 12.51
Fall	2.07 ± 1.18^abc^	13.07 ± 5.32^abc^	10.38 ± 4.83	11.57 ± 4.55	3.19 ± 1.69^a^	42.00 ± 15.85^ab^
Other	2.88 ± 1.54	17.94 ± 6.56	10.35 ± 3.37	12.94 ± 4.74	3.65 ± 2.09	48.88 ± 14.97
*F*	5.13	8.81	1.52	3.35	2.98	4.59
*P*	0.002	<0.001	0.211	0.020	0.032	0.004
Most severe site						
Head	3.67 ± 1.16	19.00 ± 1.00	15.33 ± 8.08	14.00 ± 1.73	4.00 ± 2.00	59.00 ± 12.00
Chest	2.76 ± 1.39	18.12 ± 6.09	11.04 ± 4.20	14.28 ± 4.21	3.56 ± 1.98	52.08 ± 15.31
Abdomen	2.00 ± 1.41	15.00 ± 7.96	12.50 ± 4.36	12.25 ± 4.19	3.75 ± 1.71	47.75 ± 19.07
Spine	2.14 ± 1.03	15.41 ± 4.56	10.21 ± 3.31	11.97 ± 3.67^e^	2.76 ± 1.12	44.00 ± 10.43
Pelvis	3.33 ± 1.35	20.00 ± 6.79	14.27 ± 6.46	16.47 ± 6.05^f^	4.07 ± 2.31	60.13 ± 19.45
Extremities	1.13 ± 1.25	12.67 ± 4.30^d^	9.27 ± 3.43	11.00 ± 3.89^eg^	2.80 ± 1.27	39.33 ± 12.56^g^
Lower extremities	2.30 ± 1.16	13.92 ± 5.45 ^d^	10.68 ± 4.33	11.90 ± 3.98^eg^	3.27 ± 1.69	43.74 ± 14.70
*F*	3.00	4.92	2.61	3.95	1.46	4.56
*P*	0.008	<0.001	0.019	0.001	0.192	<0.001
Type of fracture						
Single fracture	2.22 ± 1.20	13.31 ± 5.13	10.33 ± 3.98	11.37 ± 3.68	3.05 ± 1.46	41.94 ± 13.62
Multiple fractures	2.50 ± 1.24	16.72 ± 5.88	11.46 ± 4.84	13.54 ± 4.58	3.48 ± 1.88	49.65 ± 15.87
*t*	4.71	19.32	3.27	13.73	3.22	13.74
*P*	0.031	<0.001	0.072	<0.001	0.074	<0.001
Severity of injury						
Minor	2.32 ± 1.21	14.31 ± 5.49	10.56 ± 4.42	12.03 ± 4.15	3.22 ± 1.74	44.13 ± 15.10
Serious	2.63 ± 1.13	16.69 ± 4.90	11.88 ± 4.59	13.19 ± 4.19	3.59 ± 1.58	49.88 ± 13.06^h^
Devastating	2.82 ± 1.55	19.29 ± 7.50^h^	12.41 ± 4.50	15.65 ± 4.72^h^	3.18 ± 1.55	55.59 ± 16.89^h^
*F*	1.84	7.60	2.20	6.18	0.67	5.78
*P*	0.161	0.001	0.114	0.002	0.512	0.004
Activity during fracture						
No impact at al	3.67 ± 1.16	22.33 ± 4.16	17.33 ± 3.51	16.67 ± 4.04	6.33 ± 0.58	68.67 ± 8.39
A little impact	2.44 ± 1.09	15.30 ± 5.19	12.33 ± 5.11	12.78 ± 3.53	3.70 ± 1.64^i^	48.44 ± 15.16^i^
Restricted	2.09 ± 1.15^i^	13.91 ± 4.91	11.00 ± 4.77^i^	11.91 ± 3.99	3.00 ± 1.40^i^	43.82 ± 13.29^i^
Severely restricted	2.05 ± 1.13^i^	12.84 ± 4.40	9.81 ± 3.69^il^	11.81 ± 4.09	2.92 ± 1.57^il^	41.13 ± 13.26^il^
Completely inactive	2.70 ± 1.29^jk^	16.69 ± 6.35^k^	11.04 ± 4.50^i^	12.93 ± 4.69	3.36 ± 1.78^i^	48.45 ± 16.17^ik^
*F*	3.92	6.04	3.35	1.48	3.96	4.38
*P*	0.004	<0.001	0.011	0.211	0.004	0.002
Coma						
Yes	2.65 ± 1.37	18.91 ± 6.10	11.04 ± 4.03	14.65 ± 3.64	3.70 ± 1.92	52.83 ± 14.06
No	2.38 ± 1.22	14.61 ± 5.57	10.91 ± 4.54	12.24 ± 4.32	3.22 ± 1.67	45.12 ± 15.27
*t*	0.98	11.90	0.02	6.59	1.60	5.29
*P*	0.323	0.001	0.890	0.011	0.208	0.022
The degree of pain						
Mild	1.88 ± 0.99	11.54 ± 4.25	10.12 ± 4.82	11.04 ± 3.29	3.00 ± 1.55	39.23 ± 13.61
Moderate	2.31 ± 1.07	14.08 ± 4.68^m^	10.28 ± 3.90	11.68 ± 3.66	3.13 ± 1.50	43.22 ± 12.87
Severe	2.67 ± 1.39^m^	17.12 ± 6.39^mn^	11.78 ± 4.79^n^	13.74 ± 4.83^mn^	3.50 ± 1.90	50.64 ± 16.66^mn^
*F*	4.78	13.25	3.02	7.26	1.48	8.77
*P*	0.009	<0.001	0.051	0.001	0.231	<0.001

Notes: ^a^, compared with traffic accidents; ^b^, compared with fall from high; ^c^, compared with other; ^d^, compared with head; ^e^, compared with chest; ^f^, compared with spine; ^g^, compared with pelvis; ^h^, compared with minor injuries; ^i^, compared with no impact at all; ^j^, compared with restricted; ^k^, compared with severely restricted; ^l^, compared with a little impact; ^m^, compared with mild pain; ^n^, compared with moderate pain; all *P* < 0.05.

### 3.4. Factors affecting LOS by Cox's proportional-hazards regression analysis

The influencing factors selected by the Kaplan-Meier method and the indicators suggested by the previous literature that may have an impact on LOS were included in the Cox's regression model. The results showed a coefficient of *χ^2^* = 61.41 at *P* < 0.001, suggesting that the model was statistically significant. Risk factors for LOS included a serious injury (HR = 2.44, 95% *CI*: 1.33–4.46), a critical illness during hospitalization (HR = 1.70, 95% *CI*: 1.13–2.54), and undergoing 2 surgeries (HR = 1.87, 95% *CI*: 1.20–2.93) (Table 4).

**Table 4.** Cox's proportional hazards regression model analysis of influencing factors of LOS in fracture patients.

Influencing factors	*B*	*SE*	Wald χ²	*P*-value	HR	95% *CI* for HR	
						Lower	Upper
Gender^a^	0.12	0.16	0.55	0.460	1.13	0.82	1.54
Age			3.00	0.558			
≤20 years (Ref)^b^							
21–40 years	-0.43	0.56	0.59	0.442	0.65	0.22	1.94
41–60 years	-0.33	0.39	0.72	0.396	0.72	0.34	1.54
61–80 years	-0.55	0.38	2.18	0.140	0.58	0.28	1.20
>80 years	-0.45	0.37	1.54	0.215	0.64	0.31	1.30
Type of fracture^c^	-0.35	0.19	3.43	0.064	0.70	0.48	1.02
Severity of injury			8.64	0.013			
Minor (Ref)^b^							
Serious	0.89	0.31	8.30	0.004	2.44	1.33	4.46
Devastating	0.59	0.31	2.62	0.105	1.66	0.90	3.08
Critically ill	0.53	0.21	6.52	0.011	1.70	1.13	2.54
Surgery			10.45	0.015			
0(Ref)^b^							
1	0.52	0.33	2.56	0.110	1.69	0.89	3.21
2	0.63	0.23	7.65	0.006	1.87	1.20	2.93
3 and above	0.13	0.26	0.24	0.626	1.14	0.68	1.90
Admission status			5.65	0.059			
Generally (Ref)^b^							
Urgently	0.22	0.33	0.45	0.504	1.25	0.65	2.37
Critically	-0.33	0.17	3.74	0.053	0.72	0.51	1.00
History of trauma^d^	-0.12	0.17	0.52	0.470	0.88	0.64	1.23
PTSS^e^	-0.18	0.18	0.99	0.319	0.84	0.59	1.19

Notes: ^a^, (1 = male, 2 = female); ^b^, Ref: Reference for comparison; ^c^, (1 = single fracture, 2 = multiple fractures); ^d^, (1 = yes, 2 = no); ^e^, (1 = yes, 2 = no). *B*, regression coefficients; *SE*, standard error; HR, hazard ratio; *CI*, confidence interval.

### 3.5. Factors affecting PTSS by Logistic regression analysis

The independent variables that were statistically significant by the univariate analysis (*P* < 0.05) were included in the logistic regression model. The results showed that the statistically significant influencing factors were extreme activities during the fracture (OR = 0.63, 95% *CI*: 0.45–0.88) and a deepening of pain (OR = 3.34, 95% *CI*: 1.82–6.11) (Table 5).

**Table 5.** Logistic regression analysis of predictors of PTSS influencing factors.

Variable	*B*	Wald χ²	*P*-value	*OR* (95% *CI*)
Most severe site		7.77	0.255	
Head (Ref)^#^				
Chest	-1.27	0.72	0.396	0.28 (0.02–5.29)
Abdomen	-1.80	0.88	0.348	0.17 (0.00–7.08)
Spine	-2.26	2.24	0.134	0.10 (0.01–2.01)
Pelvis	-0.04	0.00	0.979	0.96 (0.05–18.19)
Upper extremities	-1.47	0.90	0.344	0.23 (0.01–4.83)
Lower extremities	-1.16	0.69	0.408	0.31 (0.02–4.90)
Type of fracture^a^	0.52	1.34	0.246	1.68 (0.70–4.01)
Severity of injury^b^	0.66	2.97	0.085	1.94 (0.91–4.10)
Activity during fracture	12.91	0.005	
No/a little impact (Ref)^#^			
Restricted	-1.76	6.18	0.013	0.17 (0.04–0.96)
Severely restricted	-1.99	11.20	0.001	0.14 (0.04–0.44)
Completely inactive	-1.82	10.00	0.002	0.16 (0.05–0.50)
Coma^c^	-0.12	0.05	0.832	0.88 (0.28–2.75)
Degree of pain^d^	1.23	13.65	0.000	3.40 (1.78–6.52)

Notes: ^#^, Ref: Reference for comparison; ^a^, (1 = single fracture, 2 = multiple fractures); ^b^, (1 = minor injuries, 2 = serious injuries, 3 = devastating injuries); ^c^, (1 = yes, 2 = no); ^d^, (1 = mild pain, 2 = moderate pain, 3 = severe pain); *B*: regression coefficients; *CI:* confidence interval.

## 4. Discussion

### 4.1. Epidemiological characteristics of hospitalized patients with traumatic fractures

The ratio of male to female patients with fractures is approximately 1.52:1, with a preponderance among the middle-aged population aged 41–60. This aligns with previous research findings which indicated a generally higher injury incidence among males compared to females [18],[19]. The gender difference may be attributed to the roles played by men and women in society and families, especially in rural areas where men, as the primary economic pillar of the household, were more frequently engaged in outdoor high-risk work. The survey further revealed that 59.31% of the patients were farmers, and approximately half had an educational level of primary school or below. Since physical labor dominates among migrant workers, their exposure to traumatic events and subsequently, the risk of injuries, was correspondingly elevated, which resulted in a higher incidence of male injuries. Additionally, our study found that the urban resident medical insurance became the primary mode of payment for medical expenses, contrary to previous research which indicated a preponderance of the New Rural Cooperative Medical Insurance [20]. This discrepancy may be attributed to recent urban-rural planning adjustments, which led to the transformation of some patients' rural household registration into urban residency [21].

This study revealed that falls are the most common cause of traumatic fractures among patients, particularly among the elderly. In non-tier-one cities in China, the incidence of falls among the elderly was relatively high, which is associated with their low physical activity levels and subsequently impacts their quality of life. Homes were the most frequent locations for fractures to occur, as the elderly faced increased risks due to inconveniences in their daily activities [22]. Additionally, accidents that involved traffic and falls from heights were prevalent among the 41–60 age group, which is potentially attributed to the lower educational levels in rural families. Some young adults with limited educational backgrounds chose to engage in high-risk occupations.

This study revealed an average ISS score of 11.56, which is slightly lower than the 12.9 reported by Warren [23] but higher than the 10.3 from domestic research [20]. Among the causes of fractures, falls typically result in minor injuries, while falls from high altitudes and traffic accidents often lead to severe injuries. Increasingly, pain is recognized as the fifth vital sign following body temperature, pulse, respiration, and blood pressure [24]. In this study, the NRS score was used to evaluate the pain of fracture patients. The average NRS score in this study was 6.79 points, which indicated a moderate pain and higher than the hip fracture study by Kornfield et al. [25]. First, it may be due to the racial differences between China and the United States. Second, our study was not limited to hip fractures after falls; therefore, the fracture trauma events involved in our study were more extensive. Studies have shown that the self-care ability scores of patients with hip fractures were 30.63 points, 53.13 points and 96.25 points when they were admitted to the hospital, first activity before surgery, and an average follow-up of 6.5 months, respectively [26]. This study found that 78.25% of patients were either partially or unable to take care of themselves when they were discharged from the hospital [27], which may be related to the time of bone recovery and clinical bone healing. Clinical experience has shown it usually takes 3-4 months or longer to fully heal after a good reduction of trauma fractures.

### 4.2. Influencing factors of LOS in inpatients with traumatic fracture

Cox's proportional-hazards regression analysis suggested that critical illness during hospitalization and surgeries were risk factors for LOS. Our study was consistent with the statistical results that the critical condition at admission and having more than 2 surgeries are the risk factors for LOS of orthopedic patients [28].

### 4.3. Influencing factors of PTSS in inpatients after traumatic fracture

In the field of orthopedic trauma, only a few studies have reported the prevalence and risk factors of PTSS, which has greatly reduced the probability of this topic being highlighted within the orthopedic literature. It's been reported that severe long-term pain and PTSS caused by major physical trauma can have significant impacts on an individual's physical and mental well-being, even leading to the occurrence of PTSD. [29]. Our study found that the positive rate of PTSS was 30.39%, which was lower than the United States (32.9%) [30] and higher than South Korea (19.5%) [31]. The reason for the difference may be the economic, social, political, and humanistic differences in various countries, which comprehensively affect the positive rate of PTSS. Our study found that pain was an influencing factor of PTSS, which was consistent with the findings of Kind et al. [32]. Psychological factors were closely related to the pain density and the disability of patients recovering from musculoskeletal trauma. Pain during hospitalization may increase the susceptibility to persistent post-traumatic stress symptoms and exceed the influence of other empirical risk factors [33]. Akhtar et al. found that the prevalence of PTSS in people with chronic pain was higher than the prevalence of PTSS in the general population (28% *vs*. 7%), and a higher pain severity was reported in chronic pain patients who were screened positive for PTSS [34].

## 5. Conclusion

The prevalence of hospitalized patients with fractures in the region and the occurrence of PTSS after fractures have been grasped through an analysis of the hospitalization data of patients with traumatic fractures. Although some influencing factors of LOS and PTSS have been discovered, there are still other risk factors, and a larger range of large sample research and analyses are needed. In order to improve the good job in preventing traumatic fractures and PTSS in the whole population, it needs the support from the government and even the whole society.

## 6. Limitations

The research data mainly originated from affiliated hospitals, which may limit its representativeness to a certain extent. Furthermore, the development of PTSD is a lengthy and intricate process. Merely focusing on acute stress characteristics may not fully reflect the true condition of PTSD. Complicating the situation, the outbreak of the COVID-19 pandemic during our recruitment process became a non-negligible variable. This sudden public health event may have caused trauma patients to face additional anxiety and other adverse emotional events, thereby exacerbating their PTSS.

## 7. Ethical review committee statement

In this study, through anonymous questionnaires, scale surveys and inpatient case data inquiries, there were no operations that were harmful to patients. In addition, the research strictly followed the principle of informed consent of the investigators, and all recovered materials were kept confidential. This study has been approved by the Institutional Review Committee of the Affiliated Hospital of Zunyi Medical University (No.[2019] 1–001).

## Use of AI tools declaration

The authors declare they have not used Artificial Intelligence (AI) tools in the creation of this article.

## References

[1] Hassanzadeh R, Farhadian M, Rafieemehr H (2023). Hospital mortality prediction in traumatic injuries patients: Comparing different SMOTE-based machine learning algorithms. BMC Med Res Methodol.

[2] Li W, Cheng P, Liu Z (2023). Post-traumatic stress disorder and traumatic events in China: a nationally representative cross-sectional epidemiological study. Psychiatry Res.

[3] Chen W, Lv H, Liu S (2027). National incidence of traumatic fractures in China: A retrospective survey of 512 187 individuals. Lancet Glob Health.

[4] Fluck D, Lisk R, Yeong K (2023). Association of polypharmacy and anticholinergic burden with length of stay in hospital amongst older adults admitted with hip fractures: A retrospective observational study. Calcif Tissue Int.

[5] Huang BX, Wang YH, Wang HB (2023). Epidemiology and the economic burden of traumatic fractures in China: A population-based study. Front Endocrinol (Lausanne).

[6] Manosroi W, Koetsuk L, Phinyo P (2023). Predictive model for prolonged length of hospital stay in patients with osteoporotic femoral neck fracture: A 5-year retrospective study. Front Med.

[7] Lv H, Chen W, Zhang T (2020). Traumatic fractures in China from 2012 to 2014: A National Survey of 512,187 individuals. Osteoporos Int.

[8] Kang KK, Ciminero ML, Parry JA (2021). The psychological effects of musculoskeletal trauma. J Am Acad Orthop Surg.

[9] Wang X, Li X, Qi M (2022). Incidence of post-traumatic stress disorder in survivors of traumatic fracture: A systematic review and meta-analysis. Psychol Health Med.

[10] American Psychiatric Association (2013). Diagnostic and statistical manual of mental disorders.

[11] Coloma-Carmona A, Carballo JL (2021). Predicting PTSS in general population during COVID-19 pandemic: The mediating role of health anxiety. J Affect Disord.

[12] Chen K, Hynes KK, Dirschl D (2024). Depression, anxiety, and post-traumatic stress disorder following upper versus lower extremity fractures. Injury.

[13] Joseph NM, Benedick A, Flanagan CD (2020). Prevalence of posttraumatic stress disorder in acute trauma patients. OTA In.

[14] National Bureau of Statistics (2010). The sixth population census.

[15] Liu X, Ma D, Liu Q (1998). Development of the post-traumatic stress disorder self-rating sca leand its reliability and validity. Chin J Behav Med Sci.

[16] Baker SP, O'Neill B, Haddon W (1974). The injury severity score: A method for describing patients with multiple injuries and evaluating emergency care. J Trauma.

[17] Baker S P, O'Neill B (1976). The injury severity score: An update. J Trauma.

[18] Hannula A, Miettinen L, Lampainen K (2023). Cost of surgical treatment for ulnar nerve entrapment in Finland, 2011–2015: A registry-based cost description study. BMJ Open.

[19] Yuan H, Guo Q, Zhang Z (2023). Sex, age, role and geographic differences in traumatic spinal fractures caused by motor vehicle collisions: A multicentre retrospective study. Sci Re.

[20] Luo B (2022). Analysis of relevant influencing factors and intervention strategies for acute stress disorder in traumatic fractures. Inner Mongolia Med J.

[21] Ren Y, Zhou Z, Cao D (2022). Did the integrated urban and rural resident basic medical insurance improve benefit equity in China?. Value Health.

[22] Audretsch CK, Siegemund A, Ellmerer A (2023). Sex differences in pelvic fractures-a retrospective analysis of 16 359 cases from the german trauma registry. Dtsch Arztebl Int.

[23] Warren AM, Jones AL, Bennett M (2016). Prospective evaluation of posttraumatic stress disorder in injured patients with and without orthopaedic injury. J Orthop Trauma.

[24] Pozza DH, Azevedo LF, Castro Lopes JM (2021). Pain as the fifth vital sign-A comparison between public and private healthcare systems. PLoS On.

[25] Kornfield SL, Lenze EJ, Rawson KS (2017). Predictors of posttraumatic stress symptoms and association with fear of falling after hip fracture. J Am Geriatr Soc.

[26] Liu J, Chen L, Duan X (2019). Effect of early preoperative mobilization on rehabilitation of the elderly patients with hip fractures after operation. Zhongguo Xiu Fu Chong Jian Wai Ke Za Zhi.

[27] Sainsbury A, Seebass G, Bansal A (2005). Reliability of the Barthel Index when used with older people. Age Ageing.

[28] Vlok M, Maloney T, Dilkes-Hall IE (2023). Reply to: Common orthopaedic trauma may explain 31,000-year-old remains. Nature.

[29] Castillo RC, Carlini AR, Doukas WC (2021). Pain, depression, and PTSD following major extremity trauma among United States military serving in Iraq and Afghanistan: Results from the METALS Study. J Orthop Trauma.

[30] Wallace M, Puryear A, Cannada LK (2013). An evaluation of posttraumatic stress disorder and parent stress in children with orthopaedic injuries. J Orthop Trauma.

[31] Lee CH, Choi CH, Yoon SY (2015). Posttraumatic stress disorder associated with orthopaedic trauma: A study in patients with extremity fractures. J Orthop Trauma.

[32] Kind S, Otis JD (2019). The interaction between chronic pain and PTSD. Curr Pain Headache Rep.

[33] Hildenbrand AK, Kassam-Adams N, Barakat LP (2020). Posttraumatic stress in children after injury the role of acute pain and opioid medication use. Pediatr Emerg Care.

[34] Akhtar E, Ballew AT, Orr WN (2019). The prevalence of post-traumatic stress disorder symptoms in chronic pain patients in a tertiary care setting: A cross-sectional study. Psychosomatics.

